# CATALYZING NOVEL METHODS OF MEASURING COGNITION BY HARNESSING TECHNOLOGY

**DOI:** 10.1093/geroni/igad104.0044

**Published:** 2023-12-21

**Authors:** Stacy Andersen, Walter Boot

## Abstract

Early detection of cognitive impairment is essential for enabling access to clinical assessments and treatments and facilitating participation in clinical trials for Alzheimer’s disease. By employing smartphones and digital technologies to capture cognitive behaviors with high precision we may be able to detect novel, subtle biomarkers of cognitive impairment during clinic-based assessments, in-home self-administered tasks, and daily activities. In this Collaborative Symposium from the Alzheimer’s Disease and Related Dementias and Technology and Aging Interest Groups, we will explore the development of technologies to detect cognitive impairment and discuss the feasibility of obtaining these data in non-traditional settings. The first presentation reports on the use of eye-tracking technology to detect cognitive impairment with high accuracy. The second presentation discusses acoustic features from digital voice recordings of a cognitive screener that are associated with cognitive impairment and increase the screener’s specificity. The next presentation describes linguistic features from voice recordings of a memory test that are associated with depression and distinct from those associated with cognitive impairment. The fourth presentation introduces a smartphone application that measures hand grip strength, a known correlate of dementia risk. The symposium concludes with a summary of older adults’ attitudes toward wearable technologies to detect cognitive decline and the development of a smart reminder system to promote adherence to home-based assessment. Overall, these presentations highlight ways we can harness technology to capture unique features of cognitive function that increase the accuracy of traditional testing or detect cognitive impairment in novel settings. This is a collaborative symposium between the Alzheimer’s Disease and Related Dementias and Technology and Aging Interest Groups.

